# A multicentre study on the incidence of respiratory viruses in children with community-acquired pneumonia requiring hospitalization in the setting of the zero-COVID policy in China

**DOI:** 10.1007/s00705-023-05698-6

**Published:** 2023-01-13

**Authors:** Ziheng Feng, Baoping Xu, Lili Zhong, Jing Chen, Jikui Deng, Zhengxiu Luo, Lingfeng Cao, Yu Tang, Changchong Li, Rong Jin, Li Deng, Yunxiao Shang, Ying Wu, Hongwei Zhao, Qianyu Feng, Xiangpeng Chen, Lili Xu, Zhengde Xie

**Affiliations:** 1 Beijing Key Laboratory of Pediatric Respiratory Infection Diseases, Key Laboratory of Major Diseases in Children, Ministry of Education, National Clinical Research Center for Respiratory Diseases, National Key Discipline of Pediatrics (Capital Medical University), Beijing Pediatric Research Institute, Beijing Children’s Hospital, Capital Medical University, National Center for Children’s Health, No. 56 Nan-li-shi Road, Beijing, 100045 China; 2 Research Unit of Critical Infection in Children, Chinese Academy of Medical Sciences, Beijing, 2019RU016 China; 3 Department of Respiratory Diseases I, Beijing Children’s Hospital, Capital Medical University, National Clinical Research Center for Respiratory Diseases, National Center for Children’s Health, Beijing, China; 4 Hunan Provincial People’s Hospital, The First Affiliated Hospital of Hunan Normal University, Changsha, China; 5 Shenyang Children’s Hospital, Shenyang, China; 6 Department of Infectious Diseases, Shenzhen Children’s Hospital, Shenzhen, China; 7 Department of Respiratory Medicine, National Clinical Research Center for Child Health and Disorders, Ministry of Education Key Laboratory of Child Development and Disorders, Chongqing Key Laboratory of Pediatrics, Children’s Hospital of Chongqing Medical University, Chongqing, China; 8 Department of Clinical Laboratory, Children’s Hospital of Fudan University, Shanghai, China; 9 Department of Respiratory, Children’s Hospital Affiliated to Zhengzhou University, Zhengzhou, China; 10 The 2nd Affiliated Hospital and Yuying Children’s Hospital of Wenzhou Medical University, Wenzhou, 325027 China; 11 Guiyang Women and Children Healthcare Hospital, Guiyang, China; 12 Guangzhou Women and Children’s Medical Center, Guangzhou, China; 13 Shengjing Hospital of China Medical University, Shenyang, China; 14 Department of Clinical Laboratory Medicine, National Children’s Medical Center, Shanghai Children’s Medical Center, School of Medicine, Shanghai Jiaotong University, Shanghai, China

## Abstract

**Background:**

Stringent nonpharmaceutical interventions (NPIs) have been implemented worldwide to combat the COVID-19 pandemic, and the circulation and seasonality of common respiratory viruses have subsequently changed. There have been few multicentre studies or comparisons of the prevalence of respiratory viruses accounting for community-acquired pneumonia (CAP) in hospitalized children between the pre-COVID period and the period after community and school reopening in the setting of the zero-COVID policy.

**Methods:**

We included 1543 children with CAP who required hospitalization from November 1, 2020 to April 30, 2021 (period 1), and 629 children with the same conditions from November 1, 2018, to April 30, 2019 (period 2), in our study. All respiratory samples from these patients were screened for six respiratory viruses (respiratory syncytial virus [RSV], adenovirus [ADV], influenza A virus [Flu A], influenza B virus [Flu B], parainfluenza virus type 1 [PIV1], and parainfluenza virus type 3 [PIV3]) using a multiplex real-time PCR assay.

**Results and conclusions:**

The median ages of the enrolled patients at the time of diagnosis were 1.5 years and 1.0 years for period 1 and period 2, respectively. In period 1, viral pathogens were detected in 50.3% (776/1543) of the enrolled patients. The most frequently identified viral pathogen was RSV (35.9%, 554/1543), followed by PIV3 (9.6%, 148/1543), PIV1 (3.6%, 56/1543), ADV (3.4%, 52/1543), Flu A (1.0%, 16/1543), and Flu B (0.8%, 13/1543). The total detection rates of these six viruses in the peak season of CAP were at the pre-COVID level. The prevalence of Flu A decreased dramatically, and circulation activity was low compared to pre-COVID levels, while the incidence of PIV3 increased significantly. There were no significant differences in the detection rates of RSV, ADV, Flu B, and PIV1 between the two periods. Our results showed that respiratory viruses accounted for CAP in hospitalized children at pre-COVID levels as communities and schools reopened within the zero-COVID policy, although the prevalence aetiology spectrum varied.

## Introduction

In response to SARS-CoV-2 (COVID-19), stringent nonpharmaceutical interventions (NPIs, e.g., mask wearing, social distancing, travel restrictions, school closures) were implemented to constrict viral spread in communities. The prevalence of common respiratory viruses varied after the COVID-19 pandemic due to NPIs and changes in health-seeking behaviours. Studies have shown a significant decrease in the detection rate of a broad spectrum of respiratory pathogens and infectious respiratory diseases attributed to community mitigation strategies [1–5]. In China, the COVID-19 pandemic was initially controlled within a few months, and communities and schools reopened in autumn 2020, which coincided with changes in COVID-19 control policies from stringent NPIs (such as lockdown and strict restriction on citizens' outdoor activity) to regular NPIs (mask wearing, social distancing, and regular COVID tests but no restriction on citizens' outdoor activity). As schools reopened, the activity of frequently detected respiratory viruses in children differed from that observed in early 2020 [6, 7].

Community-acquired pneumonia (CAP) is a significant cause of morbidity and mortality in children younger than 5 years old and imposes a huge medical and financial burden on society and families [2, 8]. Respiratory viruses are the leading cause of CAP [8–10]. There have been few real-world studies on how the prevalence of respiratory viruses in paediatric CAP patients has changed in the setting of the zero-COVID policy in China. Here, we performed a multicentre study to investigate the profile of virus activity in hospitalized children with CAP in China after community and school reopening to provide clinicians with real-world data on the viral aetiology features of these paediatric patients in the setting of the zero-COVID policy.

## Materials and methods

### Patient enrollment

From November 1, 2020, to April 30, 2021, a multicentre study aiming to profile the features of viral aetiology of CAP in hospitalized children was conducted in seven children’s hospitals or general hospitals in China, including Henan (Zhengzhou) Children's Hospital, Hunan Children’s Hospital, Shenyang Children’s Hospital, Children's Hospital of Chongqing Medical University, Shenzhen Children's Hospital, Children's Hospital of Fudan University, and Beijing Children’s Hospital. The data from November 1, 2018, to April 30, 2019, were collected from five hospitals, including Guangzhou Women and Children's Medical Center, Shengjing Hospital of China Medical University, the 2nd Affiliated Hospital and Yuying Children’s Hospital of Wenzhou Medical University, Guiyang Children’s Hospital, and Beijing Children’s Hospital. Due to the influence of stringent NPIs (such as lockdown, closure of communities, etc.) in response to the COVID-19 pandemic in early 2020, outpatient departments were closed in this period, so we were unable to collect enough data from November 1, 2019, to April 30, 2020, to include in this study.

This study included all patients between the ages of 1 month and 18 years old with a diagnosis of CAP with a disease course of respiratory symptoms not lasting more than 10 days. Patients diagnosed with CAP by meeting any of the following criteria were included: (1) Increased symptoms of cough, expectoration, or preexisting respiratory diseases and purulent sputum with or without chest pain; (2) fever; (3) signs of pulmonary consolidation and/or audible and moist rales; (4) white blood cell (WBC) count >10 × 10^9^/L or <4 × 10^9^/L, with or without nucleus left; (5) chest X-ray showing patchy, invasive, or interstitial changes with or without pleural effusion [8]. Patients with nosocomial respiratory infections (CAP diagnosed 48 hours or more after admission), inhaled foreign bodies in the airway, and acute upper respiratory infections were excluded.

### Collection of specimens and clinical data

Nasopharyngeal swabs were obtained from all enrolled children as soon as possible after admission. Pleural fluid, endotracheal aspirates, and bronchoalveolar lavage specimens that had been obtained for clinical diagnosis were also analysed. All specimens were stored in viral transport medium (VTM) at -80°C until use. Demographic, epidemiological, and clinical data were recorded by clinical researchers, using a uniform case report form (CRF).

### Molecular detection of viral pathogens

Viral nucleic acids in the clinical samples were extracted using a NucliSens easyMAG system (bioMérieux, Marcy-l’Etoile, France) according to the manufacturer’s instructions. A multiplex real-time quantitative PCR assay (XABT, Beijing, China) with high sensitivity and specificity [11] was performed for the detection of respiratory syncytial virus (RSV), adenovirus (ADV), influenza A virus (Flu A), influenza B virus (Flu B), parainfluenza virus type 1 (PIV1), and parainfluenza virus type 3 (PIV3).

### Statistical analysis

Overall detection rates and pathogen-specific detection rates were calculated from November 1, 2018, to April 30, 2019, and from November 1, 2020, to April 30, 2021. Continuous variables are presented as the mean ± standard deviation (SD) or median. Categorical variables were expressed as frequencies and percentages and compared between groups using the chi-square test or Fisher’s exact test. A *P*-value less than 0.05 was considered statistically significant. All analyses were performed using SPSS software (version 19.0 IBM Corporation, USA).

## Results

### Demographic features of patients

One thousand forty-three patients and 629 patients were enrolled from November 1, 2020, to April 30, 2021 (period 1), and November 1, 2018, to April 30, 2019 (period 2), respectively. In period 1, the median age of the enrolled patients was 1.5 years at the time of diagnosis, and 92.9% (1434/1543) of the patients were < 5 years old. In period 2, the median age of the included patients was 1.0 years, and 83.6% (526/629) of the patients were < 5 years old. The demographic features of the patients are listed in Table 1.

**Table 1:** Demographic features of children with CAP requiring hospitalization in both periods

Period	Characteristic	No. (%)
Period 1: 11/2020–4/2021	Total number of samples	1543
	Gender	
	Male	929 (60.2)
	Female	614 (39.8)
	Age	
	Median	1.5 y
	Age group	
	< 6 m	348 (22.6)
	6-12 m	282 (18.3)
	1-3 y	482 (31.2)
	3-5 y	322 (20.9)
	5-18 y	109 (7.1)
Period 2: 11/2018–4/2019	Total number of samples	629
	Gender	
	Male	374 (59.5)
	Female	255 (40.5)
	Age	
	Median	1.0 y
	Age group	
	< 6 m	205 (32.6)
	6-12 m	116 (18.4)
	1-3 y	142 (22.6)
	3-5 y	63 (10.0)
	5-18 y	103 (16.4)

### Detection of viral pathogens

Six common respiratory viruses (RSV, Flu A, Flu B, ADV, PIV1, PIV3) were detected in 50.3% (776/1543) and 47.9% (301/629) of the cases in period 1 and period 2, respectively (Table 2). There was no significant difference in the viral detection rates in the enrolled patients between period 1 and period 2 (χ^2^ = 1.1, *P* > 0.05).

**Table 2:** Positivity rates in different districts of China in both periods

District	Sample type	Positive	Negative	Positive rate (%)
***Period 1***				
Zhengzhou	NPS	88	76	53.7%
Changsha	NPS	201	176	53.3%
Shenyang	NPS	44	153	22.3%
Chongqing	NPS	162	27	85.7%
Shenzhen	NPS	79	115	40.7%
Beijing	NPS	91	142	39.1%
Shanghai	NPS	111	78	58.7%
**Total**	/	776	767	50.3%
***Period 2***				
Guangzhou	NPS	36	76	32.1%
Shenyang	NPS	31	44	41.3%
Wenzhou	NPS	117	43	73.1%
Beijing	NPS	55	92	37.4%
Guiyang	NPS	62	73	45.9%
**Total**	/	301	328	47.9%

NPS: nasopharyngeal swab

**Fig. 1:**
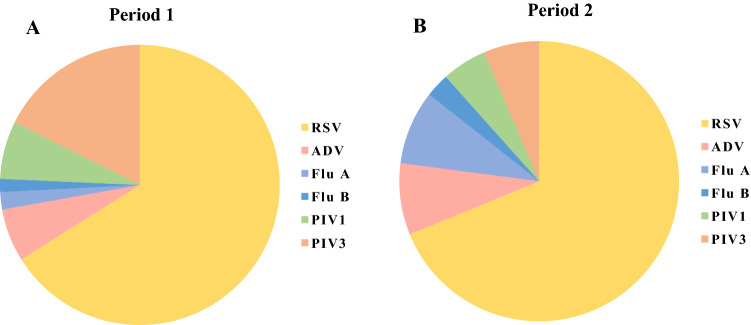
Spectrum of viral pathogens in CAP cases in both periods. (A) Spectrum of viral pathogens in CAP cases in period 1 (November 1, 2020, to April 30, 2021). (B) Spectrum of viral pathogens in CAP cases in period 2 (November 1, 2018, to April 30, 2019)

In period 1, 50.3% (776/1543) of the patients had a viral infection, and 4.1% (63/1543) of the cases were associated with coinfection. The most frequently identified viral pathogen was RSV (35.9%, 554/1543), followed by PIV3 (9.6%, 148/1543), PIV1 (3.6%, 56/1543), ADV (3.4%, 52/1543), Flu A (1.0%, 16/1543), and Flu B (0.8%, 13/1543) (Fig. 1A). RSV (90.5%, 57/63) was most frequently associated with coinfection, followed by PIV3 (57.1%, 36/63) and PIV1 (28.6%, 18/63).

In period 2, viral infection was detected in 47.9% (301/629) of cases, while 4.1% (26/629) of the patients were also coinfected with another virus. RSV (35.7%, 225/629) had the highest positivity rate, followed by Flu A (4.5%, 28/629), PIV3 (3.3%, 21/629), ADV (4.3%, 27/629), PIV1 (2.7%, 17/629), and Flu B (1.4%, 9/629) (Fig. 1B). RSV (80.8%, 21/26) was the most frequently detected virus in coinfection cases, followed by ADV (38.5%, 10/26) and Flu A (30.8%, 8/26).

**Fig. 2:**
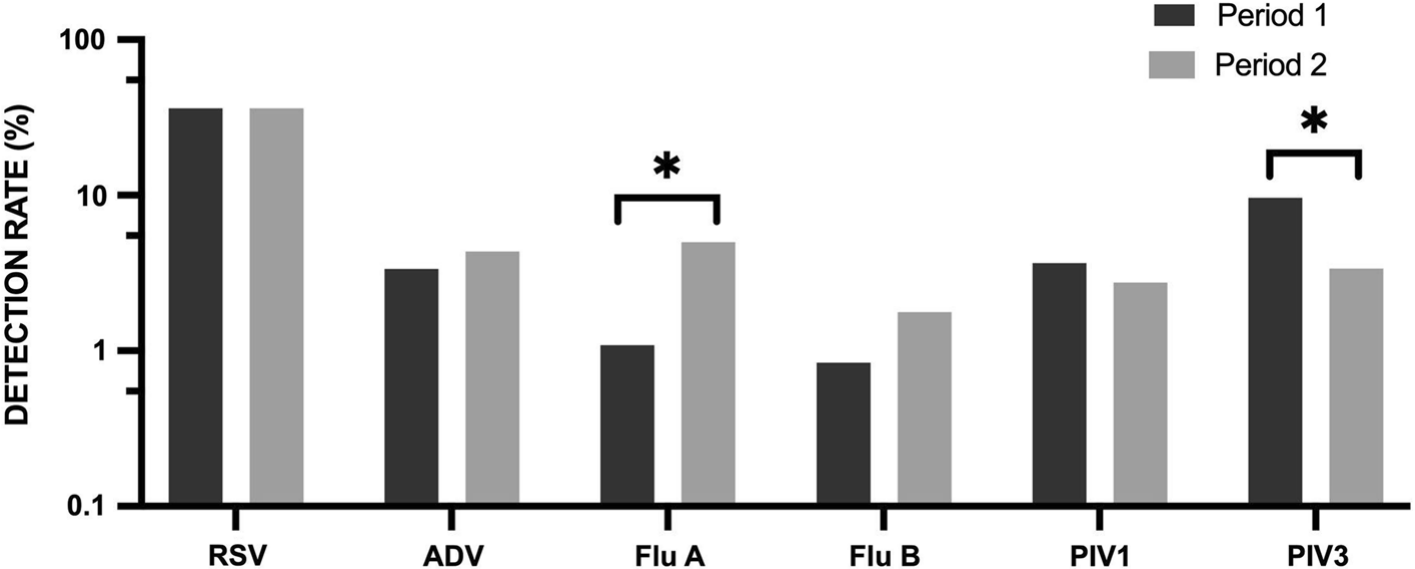
Comparison of the rate of detection of each virus between the two periods. *indicates a statistically significant difference in the detection rate between the two periods using the chi-square test or Fisher’s exact test. A *P*-value less than 0.05 was considered statistically significant.

Comparing the detection rates for each virus between these two periods, no significant difference was observed in the detection rates for RSV, ADV, Flu B, and PIV1. However, the incidence of Flu A during the later period had decreased from 4.5% to 1% (χ^2^ = 26.3, *P* < 0.001). By contrast, the incidence of PIV3 during the later period had increased from 3.3% to 9.6% (χ^2^ = 24.4, *P* < 0.001) (Fig. 2).

### Comparison of the rates of detection of viruses in different age groups between the two periods

**Fig. 3:**
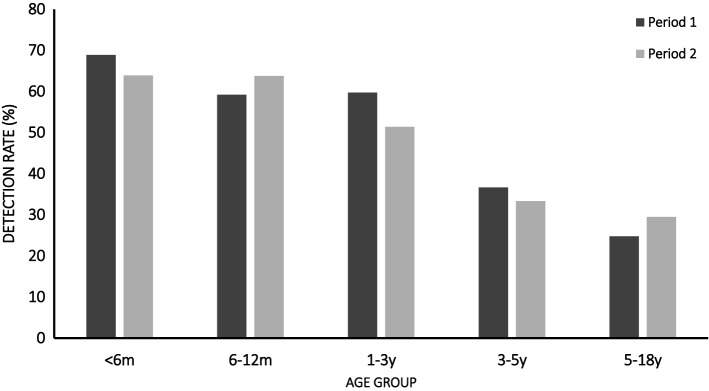
Comparison of detection rates in different age groups between the two periods

In both periods, the positivity rates of the viruses decreased as age increased (period 1, χ^2^ = 116.3, *P* < 0.001; period 2, χ^2^ = 52.3, *P* < 0.001). The highest rate of detection of viruses was in the <6 months age group. Within the individual age groups, no significant difference in the detection rate was observed between period 1 and period 2 (Fig. 3).

The distribution of each virus by age group is presented in Figure 5. RSV had the highest positivity rate in all age groups in both periods. We observed that the Flu A positivity rate was lower in all age groups and that the PIV3 positivity rate was higher in all age groups in period 1 than in period 2.

The detection rates of viral pathogens in different age groups between these two periods are listed in Table 3. The detection rates of RSV, Flu B, and PIV1 were the same as those before COVID-19. The detection rate of Flu A was statistically significantly lower in the 6-12 months (*P* < 0.001) and 1-3 years old (*P* = 0.011) age groups. ADV was less frequently detected in period 1 than in period 2 in the <6 months (*P* = 0.006) age group. However, the detection rate of PIV3 was statistically significantly higher in the <6 months (from 3.9% to 12.1%, *P* = 0.003), 6-12 months (from 5.2% to 10.6%, *P* = 0.048), 3-5 years (from 0.0% to 7.8%, *P* = 0.013), and 5-18 years (from 0.0% to 4.6%, *P* = 0.023) age groups.

**Table 3:** Detection rates of six respiratory viruses in different age groups

Virus and age group	Period 1 (no. %)	Period 2 (no. %)	*P*-value
**RSV**			
<6 m	177 (50.9)	99 (48.3)	-
6-12 m	119 (42.2)	47 (40.5)	-
1-3 y	188 (39.0)	43 (30.3)	-
3-5 y	58 (18.0)	16 (25.4)	-
5-18 y	12 (11.0)	20 (19.4)	-
**ADV**			
<6 m	6 (1.7)	13 (6.3)	*P* = 0.006
6-12 m	5 (1.8)	5 (4.3)	-
1-3 y	19 (3.9)	6 (4.2)	-
3-5 y	18 (5.6)	1 (1.6)	-
5-18 y	4 (3.7)	2 (1.9)	-
**Flu A**			
<6 m	4 (1.1)	4 (2.0)	-
6-12 m	3 (1.1)	11 (9.5)	*P* < 0.001
1-3 y	7 (1.5)	8 (5.6)	*P* = 0.011
3-5 y	2 (0.6)	1 (1.6)	-
5-18 y	0 (0.0)	4 (3.9)	-
**Flu B**			
<6 m	3 (0.9)	2 (1.0)	-
6-12 m	1 (0.4)	1 (0.9)	-
1-3 y	4 (0.8)	4 (2.8)	-
3-5 y	3 (0.9)	1 (1.6)	-
5-18 y	2 (1.8)	1 (1.0)	-
**PIV1**			
<6 m	7 (2.0)	5 (2.4)	-
6-12 m	9 (3.2)	4 (3.4)	-
1-3 y	24 (5.0)	5 (3.5)	-
3-5 y	12 (3.7)	2 (3.2)	-
5-18 y	4 (3.7)	1 (1.0)	-
**PIV3**			
<6 m	42 (12.1)	8 (3.9)	*P* = 0.003
6-12 m	30 (10.6)	6 (5.2)	*P* = 0.048
1-3 y	46 (9.5)	7 (4.9)	-
3-5 y	25 (7.8)	0 (0.0)	*P* = 0.013
5-18 y	5 (4.6)	0 (0.0)	*P* = 0.023

–, no statistically significant difference

### Characteristics of viral pathogens in North and South China during the two periods

**Fig. 4:**
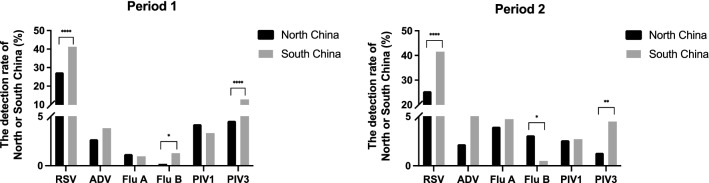
Comparison of detection rates in different areas between the two periods. *indicates a statistically significant difference in the detection rate between the two periods. *, *P* < 0.05; **, *P* < 0.01; ***, *P* < 0.001; ****, *P* < 0.0001

Next, we analyzed the difference in detection rates between North China and South China during the two periods. We defined Henan, Shenyang, Beijing in period 1 and Shenyang and Beijing in period 2 as North China, while Hunan, Chongqing, Shenzhen, Shanghai in period 1 and Guizhou, Guangzhou, and Wenzhou in period 2 were defined as South China. RSV was the most frequently detected pathogen in North and South China in both periods, and the detection rate in South China was significantly higher than that in North China (Fig. 4). The incidence of PIV3 in South China was also significantly higher than that in North China in both periods (Fig. 4). The detection rate of Flu B in different areas showed different patterns in the two periods, when its detection rate was significantly higher in North China during period 2 but higher in South China during period 1 (Fig. 4).

There were no significant differences in the overall viral rate of detection in enrolled patients in North or South China between the two periods (in North China, period 1, 40.2%, period 2, 38.8%, χ^2^ = 0.1479, *P* > 0.05; in South China, period 1, 63.2%, period 2, 59.5%, χ^2^ = 1.707, *P* > 0.05). For Flu A, the incidence of which was significantly lower in period 1, the detection rate was significantly lower in both areas of China during period 1 than during period 2 (Table 4). For PIV3, which was detected more frequently in period 1, the detection rate was significantly higher in both areas during period 1 than during period 2 (Table 4).

**Table 4:** Detection rates of six respiratory viruses in North China and South China

Areas	Virus	Period 1 (no. %)	Period 2 (no. %)	*P*-value
North China	RSV	163 (27.4)	58 (25.6)	-
	ADV	16 (2.7)	5 (2.2)	-
	Flu A	7 (1.2)	9 (4.0)	0.0098
	Flu B	1 (0.2)	7 (3.1)	0.0001
	PIV1	25 (4.2)	6 (2.6)	-
	PIV3	27 (4.5)	3 (1.3)	0.0277
South China	RSV	391 (41.2)	167 (41.5)	-
	ADV	36 (3.8)	22 (5.5)	-
	Flu A	9 (0.9)	19 (4.7)	< 0.0001
	Flu B	12 (1.3)	2 (0.5)	-
	PIV1	31 (3.3)	11 (2.7)	-
	PIV3	121 (12.8)	18 (4.5)	< 0.0001

–: no statistically significant difference

## Discussion

Pneumonia is responsible for a great proportion of morbidity and mortality in children [8]. Previous studies have shown that the circulation of respiratory viruses decreased after stringent NPI implementation to eliminate community transmission of COVID-19 in both the Northern and Southern Hemispheres [1, 3, 4]. In China, studies have shown that the circulation activity and detection rate of common respiratory viruses accounting for CAP in hospitalized children decreased sharply during lockdown and school closure [7, 12]. Here, we performed a retrospective observational multicentre study to determine the viral aetiology with CAP in children requiring hospitalization before and after the COVID-19 pandemic outbreak. In our study, we used a multiplex real-time PCR assay to detect RSV, ADV, Flu A, Flu B, PIV1, and PIV3 in hospitalized children with CAP after community and school reopening within the setting of the zero-COVID policy. The results showed that the overall detection rates of these six viruses in the peak season of CAP were at pre-COVID levels. There were no significant differences in the detection rates of RSV, ADV, Flu B, and PIV3 between period 1 and period 2. These data might indicate that the activity of some respiratory viruses returned to pre-COVID levels after communities and schools reopened, even in the setting of the zero-COVID policy.

RSV was the most frequently detected pathogen in all age groups in both periods, with the greatest burden in children less than 3 years old. The positivity rate of RSV in children less than 3 years of age was the highest, which accounted for a large proportion of the total detection rate. Children less than 2 years of age were most susceptible to RSV infection [13]. There was no significant difference in the positivity rate of RSV between the two periods. Other studies have indicated a resurgence of RSV after community reopening [6, 7, 14]. Although NPIs, including mask wearing and social distancing, are still maintained in China after community reopening, children younger than 3 years of age may be reluctant to wear masks, and improperly sized masks may not provide enough protection.

In our study, the detection rate of influenza A virus decreased dramatically, and circulation was low compared to pre-COVID levels. Influenza virus mainly spreads through the population via large droplets, small particle aerosols, and direct or indirect contact, and mask wearing (surgical masks, KN95 masks and N95 masks) as well as social distancing could effectively reduce the outward particle emission of influenza virus [15–17]. Stringent mask wearing and social distancing were still maintained after the COVID-19 pandemic was under control in China, which could reduce circulation among adults and subsequently reduce household transmission. Influenza virus has been characterized as a cause of travel-related disease [18], and studies have shown that air travel plays an important role in the long-range dissemination of influenza [19]. Restrictions on domestic and international travel could contribute to the low circulation of influenza A virus. Although the NPIs in communities have been eased compared to early 2020, strict COVID-19 controls remain in effect in hospitals to prevent nosocomial transmission of SARS-CoV-2. Thus, changes in health-seeking behaviours in the setting of the zero-COVID policy might have contributed to the low rate of detection of Flu A. Meanwhile, RSV and PIV3 mainly cause symptoms such as croup and wheezing [13, 20], making parents more likely to take their children to the hospital. Therefore, the low prevalence observed with Flu A was not observed with RSV and PIV3. Furthermore, viral-viral interactions might have been a factor contributing to the low rate of detection of Flu A. Studies have shown that rhinovirus circulation remains at high levels after stringent NPIs and could trigger interferon-stimulating immunity, and this might hamper influenza virus infection in the population [21]. Despite this, vaccination of individuals over 6 months of age should continue throughout the influenza season to protect them from severe health consequences [22].

Notably, the rate of detection of PIV3 increased significantly compared to the pre-COVID-19 level. Another study showed similar results [7]. PIV3 infections usually occur in spring and early summer each year [23, 24]. The buildup of susceptibility resulting from stringent NPIs in early 2020 might have influenced the seasonality of PIV3 and resulted in larger outbreaks on average outside the peak season [25]. The exact reason for this is unclear, and studies on the molecular epidemiology of PIV3 are needed to determine whether mutations in PIV3 caused the increase in its detection rate. In either case, this result suggests that health care systems should prepare for possible PIV3 outbreaks in early 2022.

There are several limitations to our study. This is a multicentre study, but the cooperation between hospitals and our study was interrupted due to the COVID-19 pandemic in period 2; thus, we included samples from seven different hospitals in period 1. However, the hospitals where samples were collected in period 1 or in period 2 represent different areas of China (northeast, northwest, southeast, southwest, and central). Meanwhile, there was a shortage of data from November 1, 2019, to April 30, 2020, in this study because the COVID-19 pandemic broke out in January 2020 in China, and the majority of laboratories prioritized testing for COVID-19. Additionally, lockdown resulted in a significant decrease in outpatient visits and admissions. Furthermore, in this study, we tested for only six common viral pathogens in respiratory samples from children with CAP. Other viral pathogens and causal agents, including bacteria and mycoplasma, need to be investigated in future studies. In addition, outpatient children with CAP were not included in this study, so our findings may not represent the overall population of children.

In conclusion, we observed that respiratory viruses accounted for CAP in hospitalized children at pre-COVID levels in the setting of the zero-COVID policy, although the prevalence aetiology spectrum varied. The incidence of influenza A virus decreased dramatically, and circulation was low compared to pre-COVID levels, while the incidence of PIV3 increased significantly. Given that children are the last to access novel vaccines and susceptibility built up during the COVID-19 control period, strategies are needed to protect children from emerging viruses and common respiratory viral pathogens.

## Data Availability

The datasets used and/or analyzed during the current study are available from the corresponding author on reasonable request.
